# Transcriptome Analyses Reveal the Involvement of Both C and N Termini of Cryptochrome 1 in Its Regulation of Phytohormone-Responsive Gene Expression in *Arabidopsis*

**DOI:** 10.3389/fpls.2016.00294

**Published:** 2016-03-14

**Authors:** Wen-Xiu Wang, Hong-Li Lian, Li-Da Zhang, Zhi-Lei Mao, Xiao-Ming Li, Feng Xu, Ling Li, Hong-Quan Yang

**Affiliations:** ^1^Key Laboratory of Urban Agriculture (South) Ministry of Agriculture and School of Agriculture and Biology, Shanghai Jiao tong UniversityShanghai, China; ^2^State Key Laboratory of Genetic Engineering and Collaborative Innovation Center for Genetics and Development, School of Life Sciences, Institute of Plant Biology, Fudan UniversityShanghai, China

**Keywords:** CRY1, CRY1 C terminus (CCT1), CRY1 N terminus (CNT1), RNA-Seq, gibberellin acids (GA), brassinosteroids (BR), auxin

## Abstract

Cryptochromes (CRY) are blue-light photoreceptors that mediate various light responses in plants and animals. It has long been demonstrated that *Arabidopsis* CRY (CRY1 and CRY2) C termini (CCT1 and CCT2) mediate light signaling through direct interaction with COP1. Most recently, CRY1 N terminus (CNT1) has been found to be involved in CRY1 signaling independent of CCT1, and implicated in the inhibition of gibberellin acids (GA)/brassinosteroids (BR)/auxin**-**responsive gene expression. Here, we performed RNA-Seq assay using transgenic plants expressing CCT1 fused to β-glucuronidase (*GUS-CCT1*, abbreviated as *CCT1*), which exhibit a constitutively photomorphogenic phenotype, and compared the results with those obtained previously from *cry1cry2* mutant and the transgenic plants expressing CNT1 fused to nuclear localization signal sequence (NLS)-tagged YFP (*CNT1-NLS-YFP*, abbreviated as *CNT1*), which display enhanced responsiveness to blue light. We found that 2903 (67.85%) of the CRY-regulated genes are regulated by CCT1 and that 1095 of these CCT1-regulated genes are also regulated by CNT1. After annotating the gene functions, we found that CCT1 is involved in mediating CRY1 regulation of phytohormone-responsive genes, like CNT1, and that about half of the up-regulated genes by GA/BR/auxin are down-regulated by CCT1 and CNT1, consistent with the antagonistic role for CRY1 and these phytohormones in regulating hypocotyl elongation. Physiological studies showed that both CCT1 and CNT1 are likely involved in mediating CRY1 reduction of seedlings sensitivity to GA under blue light. Furthermore, protein expression studies demonstrate that the inhibition of GA promotion of HY5 degradation by CRY1 is likely mediated by CCT1, but not by CNT1. These results give genome-wide transcriptome information concerning the signaling mechanism of CRY1, unraveling possible involvement of its C and N termini in its regulation of response of GA and likely other phytohormones.

## Introduction

Light not only provides plants with energy to maintain life, but also supplies plants with signal to regulate their developmental processes in the whole life cycle (Chen et al., 2004). Plants have evolved various photoreceptors including blue/UV-A light receptors cryptochromes (CRY1 and CRY2), phototropins (PHOT1 and PHOT2) (Cashmore et al., 1999; Briggs and Christie, 2002), the red/far-red light receptors phytochromes (phyA to phyE) (Quail et al., 1995; Quail, 2002), and the UV-B receptor UVR8 (Rizzini et al., 2011). It is known that CRY1 and CRY2 regulate a variety of physiological processes (Ahmad and Cashmore, 1993; Guo et al., 1998; Somers et al., 1998; Mao et al., 2005; Kang et al., 2009), including seedling photomorphogenesis and photoperiodic flowering, respectively. CRY contains an N-terminal domain related to the photolyase and a C-terminal extension domain (Yang et al., 2000; Sang et al., 2005). The C-termini of CRY1 and CRY2 (CCT1 and CCT2) are known to mediate CRY1 signaling, since transgenic plants expressing CCT1 or CCT2 fused to β-glucuronidase (GUS) show a constitutively photomorphogenic phenotype (Yang et al., 2000). It is demonstrated that the N termini of CRY1 and CRY2 (CNT1 and CNT2) are not only responsible for chromophores binding and blue light perception (Lin et al., 1995; Lin, 2002), but also mediation of their dimerization, which is essential for light activation of their photoreceptor activity (Sang et al., 2005; Yu et al., 2007). Most recently, CNT1 has been shown to mediate CRY1 signaling independent of its C terminus, since CNT1 fused to nuclear localization signal sequence (NLS)-tagged YFP confers enhanced responsiveness to blue light (He et al., 2015).

Several CRY-interacting proteins have been characterized. Of them, the master negative regulator of photomorphogenesis, COP1 (CONSTITUTIVELY PHOTOMORPHOGENIC1) (Osterlund et al., 1999), is the first characterized, with which CRY1 or CRY2 interacts through their C termini (Yang et al., 2000, 2001; Wang et al., 2001). COP1 has E3 ubiquitin ligase activity, and physically interacts with various substrates, such as HY5/HYH, HFR1, LAF1, and CONSTANS, to promote their ubiquitination and degradation to regulate photomorphogenesis and flowering (Holm et al., 2002; Seo et al., 2003; Yang et al., 2005; Liu et al., 2008b). Among them, HY5, a bZIP transcription factor, plays a pivotal role in promoting photomorphogenesis (Osterlund et al., 2000). Recent studies have revealed that CRY1 and CRY2 interact with COP1's E3 ubiquitin ligase enhancer, SPA1 (SUPPRESSOR OFPHYTOCHROME A 1), through their C and N termini, respectively (Lian et al., 2011; Liu et al., 2011; Zuo et al., 2011). The interactions of CRY1 with COP1 and SPA1 result in the dissociation of the COP1-SPA1 complex and eventually the accumulation of HY5 protein (Lian et al., 2011; Liu et al., 2011). CRY2 is also found to interact with a family of bHLH transcription factors, cryptochrome-interacting bHLH (CIBs), through its N terminus, to regulate flowering (Liu et al., 2008a; Kennedy et al., 2010). To date, no CRY1 N terminus-interacting protein has been identified.

Several phytohormones, such as gibberellin acids (GA), brassinosteroids (BR), and auxin, are involved in promoting hypocotyl cell elongation. The biosynthesis mutants of GA, BR, and auxin, as well as of the loss-of-function mutants of GA receptor, GID1, BR receptor, BRI1, and auxin receptors, TIR1/AFBs, show a shortened hypocotyl phenotype during photomorphogenic development (Chory et al., 1991; Clouse et al., 1996; Kim et al., 1996; Szekeres et al., 1996; Richards et al., 2001; Dharmasiri et al., 2005; Ueguchi-Tanaka et al., 2005). The antagonistic regulation of hypocotyl cell elongation by light and these phytohormones indicates that the signaling mediated by light and these phytohormones may antagonize. Indeed, it has been demonstrated that the signaling crosstalk between GA and light is mediated through repression of the transcriptional activity of Phytochrome-Interacting Factors 3 and 4 (PIF3 and PIF4), pivotal negative regulators of photomorphogenesis (Leivar et al., 2008), through direct interactions of DELLA proteins with PIF3 and PIF4 (de Lucas et al., 2008; Feng et al., 2008). In addition, brassinosteroid and light can be converged through the interaction of PIF4 with BZR1, a critical transcription factor acting togerther with BES1 to mediate BR response (Oh et al., 2012). HY5 is also shown to be involved in regulating auxin, GA, and BR signaling through regulating genes involved in phytohormone biosynthesis or signaling (Cluis et al., 2004; Weller et al., 2009; Li and He, 2016). The newly identified branch inhibiting phytohormone, strigolactones (Jiang et al., 2013; Zhou et al., 2013), regulates hypocotyl elongation through promoting HY5 protein accumulation, which is dependent on cryptochrome and phytochrome signaling pathways (Jia et al., 2014).

It has been demonstrated that cryptochromes regulate GA catabolic/metabolic genes to inhibit the biosynthesis of active GA and thus hypocotyl elongation (Zhao et al., 2007). A recent study shows that CNT1 is likely involved in mediating CRY1 repression of GA/BR/auxin-responsive gene expression (He et al., 2015). Given that CNT1 and CCT1 are both involved in mediating CRY1 signaling, it is worth investigating how they are involved in regulating phytohormone-responsive gene expression at the transcriptomic level. In this study, we performed RNA-Seq assay using transgenic plants expressing CCT1 fused to β-glucuronidase (GUS) (*GUS-CCT1*, abbreviated as *CCT1*), and compared with the RNA-Seq results we obtained from *cry1cry2* and *CNT1* seedlings previously (He et al., 2015). We uncovered 6780 CCT1-regulated genes, a portion of which are CRY-regulated GA/BR/auxin-responsive genes, indicating the possible involvement of CCT1 in mediating CRY1 regulation of phytohormone-responsive genes. CCT1 and CNT1 not only co-regulate, but also separately regulate some of the GA/BR/auxin-responsive genes that are regulated by CRY. The results of physiological studies show that both CCT1 and CNT1 are likely involved in mediating CRY1 reduction of seedlings sensitivity to GA under blue light. Protein expression studies indicate that CCT1, but not CNT1, may be involved in mediating CRY1 inhibition of GA-promoted degradation of HY5, indicating possible different mechanisms for CCT1 and CNT1 in mediating CRY1 regulation of GA response. Hence, our study gives new insight into the signaling mechanism of CRY1, providing clue to looking for potential downstream components of CRY1 that may interact with CCT1 or CNT1.

## Materials and methods

### Plant materials and growth conditions

*Arabidopsis thaliana* ecotype Columbia (Col-0) was used as the wild type (WT) control. The transgenetic line overexpressing CCT1 fused to β-glucuronidase (GUS) (*GUS-CCT1*, abbreviated as *CCT1*), transgenic *cry1* mutant line #9 overexpressing CNT1-NLS-YFP (abbreviated as *CNT1*), the seedlings overexpressing MYC-tagged full-length CRY1 (*CRY1-ovx*), *cop1-4*, and *cry1* mutant were described previously and all in the Col-0 background (Yang et al., 2000; Mao et al., 2005; He et al., 2015). Imbibed seeds were kept for 3 days at 4°C and grown on half-strength Murashige-Skoog (MS) nutrient medium plus 1% sucrose with 0.8% agar at 22°C under white light (100 μmol/m^2^/s) or 30 μmol/m^2^/s blue light.

### Rna extraction, library preparation, and sequencing

Seeds were germinated on half-strength MS plates plus 1% sucrose and placed at 4°C for 3 days and then transferred to white light for 12 h before placed in darkness for another 4 days. Two biological replicates were prepared for WT, *CCT1* and *cop1-4* plants. Total RNA were extracted with RNAprep plant kit (TIANGEN) and treated with DNase I (TIANGEN) following the manufactuer's instruction. Quality control was performed with Agilent 2100 Bioanalyzer. The cDNA libraries were constructed using NEBNextUltra™ RNA library Prep Kit and submitted for sequencing using Illumina Hiseq2500. The library construction and sequencing were performed by the Hanyu BioTech in Shanghai (Pu Dong, Shanghai, China).

### Processing of RNA-seq data

Raw sequencing reads were processed with FASTX-Toolkit (v0.0.13) to trim adaptor contaminations and filter out low quality reads with default parameters. The processed reads were then mapped to the *Arabidopsis* TAIR10 genome assembly using Bowtie 2.2.2 with default parameters (Langmead and Salzberg, 2012). The mapped reads for each gene were counted with samtool and then converted into RPKM (Mortazavi et al., 2008). The MARS (MA-plot-based method with Random sampling model) from DEGseq package was used to call significant differentially expressed genes (DEGs) (Wang et al., 2010). When identifying differentially expressed genes between two samples, we considered both fold change and *p*-value. *P*-value was calculated for each gene with expression values obtained from RNA-Seq analysis being subjected to two-tailed unpaired Student *t*-test. Genes with a fold change >2 and *p* < 0.01 were defined as differentially expressed genes. Venn diagram was generated in Venny (http://bioinfogp.cnb.csic.es/tools/venny/index.html). Heat-map was generated with hierarchical clustering analysis by MeV 4.7 software (He et al., 2015).

### Function and pathway enrichment analysis

The Gene Ontology (GO) enrichment analysis is based on the GOseq method (Young et al., 2010), which is based on Wallenius non-central hyper-geometric distribution. We identified the significantly enriched GO term of DEGs with FDR (*q*-value) < 0.05.

For KEGG analysis, we used KEGG pathway as a unit and applied the hypergeometric test to find significant enriched pathways compared to the whole genome background (Kanehisa and Goto, 2000). We identified the significantly enriched KEGG pathway of DEGs with FDR (*q*-value) < 0.05. Differential gene KEGG enrichment and scatter plot is a graphical display of the results of KEGG enrichment analysis. Rich Factor means the number of genes that are differentially expressed in the pathway entries and the ratio of the total number of genes located in the pathway entries to all of the annotated genes.

### Real-time quantitative PCR

We used WT, *cry1* mutant, *CRY1-ovx, CCT1*, and *CNT1* seedlings for real-time quantitative PCR. Seedlings were germinated on half-strength MS plates plus 1% sucrose and placed at 4°C for 3 days and then transferred to white light for 12 h before all seedlings placed in darkness for another 4 days. Then half of the seedlings were exposure to 30 μmol/m^2^/s blue light for 1 h, and another half of the seedlings were continue grown in dark. Total RNAs were isolated with RNAprep Plant kit (TIANGEN) followed by DNase I (TIANGEN) treatment. Then 500 ng sample of total RNA were used to reverse-transcribed to 10 μl cDNA using iScriptcDNA Synthesis kit (Bio-Rad). To validate our expression profile data, we selected genes that showed significant expression changements according to our different aim and performed real-time quantitative PCR. qRT-PCR was described previously (Zhang et al., 2014; He et al., 2015) and ACT2 was used as internal control for qRT-PCR. The used primers are listed in Table S4.

### GA and PAC treatment

GA_3_ and PAC stocks were prepared in ethanol. WT, *cry1* mutant, *CRY1-ovx, CCT1*, and *CNT1* seedlings were grown on half-strength MS plates supplementing gradient GA (mock, 0.05, 0.2, 0.5, 1.0 μM), or PAC concentrations (mock, 0.025, 0.05, 0.2, 0.5 μM), or PAC (1 μM) plus GA concentrations (0.0, 0.5, 1.0, 2.5, 5.0, 10.0 μM) at 4°C for 3 days, then placed in white light overnight before transferred into blue light for another 5–6 days. Seedlings were neatly stacked on the agar plates. Then seedlings were photographed and the hypocotyl length was measured with Image J software.

### Western blotting

WT, *cry1* mutant, *CRY1-ovx, CCT1*, and *CNT1* seedlings were grown in plates treated with gradient GA concentrations (mock, 1, 2.5 μM), PAC (mock, 0.5, 1 μM) at 4°C for 3 days, then placed in white light overnight before transferred into blue light for another 4 days. Seedlings were collected and homogenized in lysis buffer (50 mM Tris-HCl pH7.5, 150 mM NaCl, 1 mM EDTA, 10% glycerol, 0.2% Trition-X-100, 1 mM Pefabloc, cocktail, 50 μM MG132). The protein were quantified with Bradford assay (Bio-Rad), and subjected to Western blotting analyses with anti-HY5 antibody (Jia et al., 2014), and anti-ACT11 antibody (Abmart), respectively. The *cop1-4* mutant served as a positive control.

### Accession numbers

All RNA-seq data have been submitted to the NCBI Sequence Read Archive (SRA) (http://www.ncbi.nlm.nih.gov/sra/) under the accession number SRP067949. The accession numbers of previous microarray or RNA-Seq datasets used in this study are GSE58552 and GSE59763 (He et al., 2015), GSE863 (auxin/mock) (Nemhauser et al., 2006), GSE51772 (BR/mock) (Oh et al., 2014), and GSE22681 (*ga1-3*/WT) (Cheminant et al., 2011).

## Results

### Identification of genes regulated by CCT1

It has been reported recently that CRY1 N terminus (CNT1) mediates CRY1 signaling independent of its C terminus (CCT1), and CNT1 influenced about one third of the CRY-regulated genes (He et al., 2015). To gain more information concerning the signaling mechanism of CRY1 mediated by its C terminus at the transcriptomic level, we performed RNA-Seq analysis with WT and the transgenic plants expressing CCT1 fused to β-glucuronidase (GUS) (*CCT1*) through the Illumina HiSeq2500 system. A total of 22,791,289 and 25,743,501 successful sequencing reads were produced for WT and *CCT1* plants, respectively (Table 1). Notably, more than 90.56 and 70.39% of the reads for WT and *CCT1* were mapped to the *Arabidopsis* genome (version 10), and 84.18 and 63.81% reads were uniquely mapped to a single location in WT and *CCT1*, respectively (Table 1). Furthermore, 6780 differentially expressed genes (DEGs) between WT and *CCT1* were identified with a *p* < 0.01 and fold change >2 (Table S1). In this study, we primarily focused on the DEGs.

**Table 1:** **Summary of RNA-Seq data from WT and *CCT1***.

**Map to gene**	**CCT1_D1**		**CCT1_D2**		**WT_D1**		**WT_D2**	
	**Reads number**	**Percentage (%)**	**Reads number**	**Percentage (%)**	**Reads number**	**Percentage (%)**	**Reads number**	**Percentage (%)**
Clean reads	22791286	100	27312797	100	25743501	100	22651750	100
Total mapped reads	16041945	70.39	18807037	68.86	23312549	90.56	20382779	89.98
Unique_match	14543021	63.81	17086853	62.56	21670195	84.18	18921019	83.53
Perfect match	15022315	65.91	17613248	64.49	21919460	85.15	19150500	84.54

### Functional enrichment analysis indicates CCT1 involvement in CRY1 regulation of phytohormone-responsive gene expression

**Figure 1:**
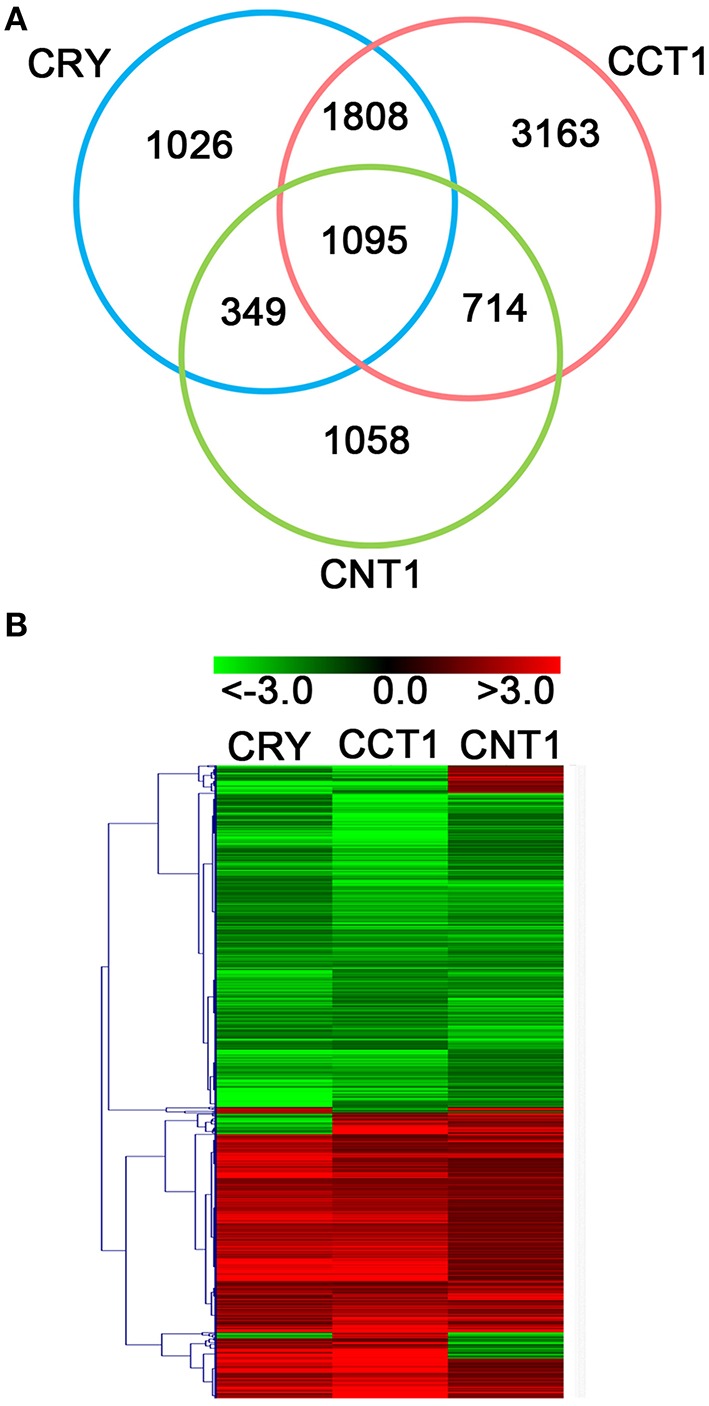
**CCT1, CNT1, and CRY regulate a large number of overlapping genes in the same direction**. **(A)** Venn diagram showing significant overlapping genes between CCT1, CNT1, and CRY datasets. **(B)** Hierarchical clustering analysis of 1095 overlapping genes as shown in **(A)**. Red and green colors in the heat-maps represent induced and repressed genes, respectively. Scale bar denotes the log_2_ value of fold change.

Since both CCT1 and CNT1 mediate CRY1 signaling (Yang et al., 2000; He et al., 2015), and CRY1 N terminus is involved in the regulation of growth-related phytohormone-responsive gene expression, we examined whether CCT1 might also be involved in mediating CRY1 regulation of phytohormone-responsive genes. Firstly, we examined how many of the CRY-regulated genes might be regulated by CCT1. Notably, CCT1 regulated 2903 (67.85%) of the 4278 CRY-regulated genes in the same direction (Figures 1A,B). Venn diagram and Heat-map graph revealed that, of the 2903 CCT1-regulated genes, 1095 were also regulated by CNT1 in the same direction (Figures 1A,B), indicating that the overlapping is statistically significant. These results suggested that CCT1 is involved in mediating CRY1 signaling at the transcriptomic level.

**Figure 2:**
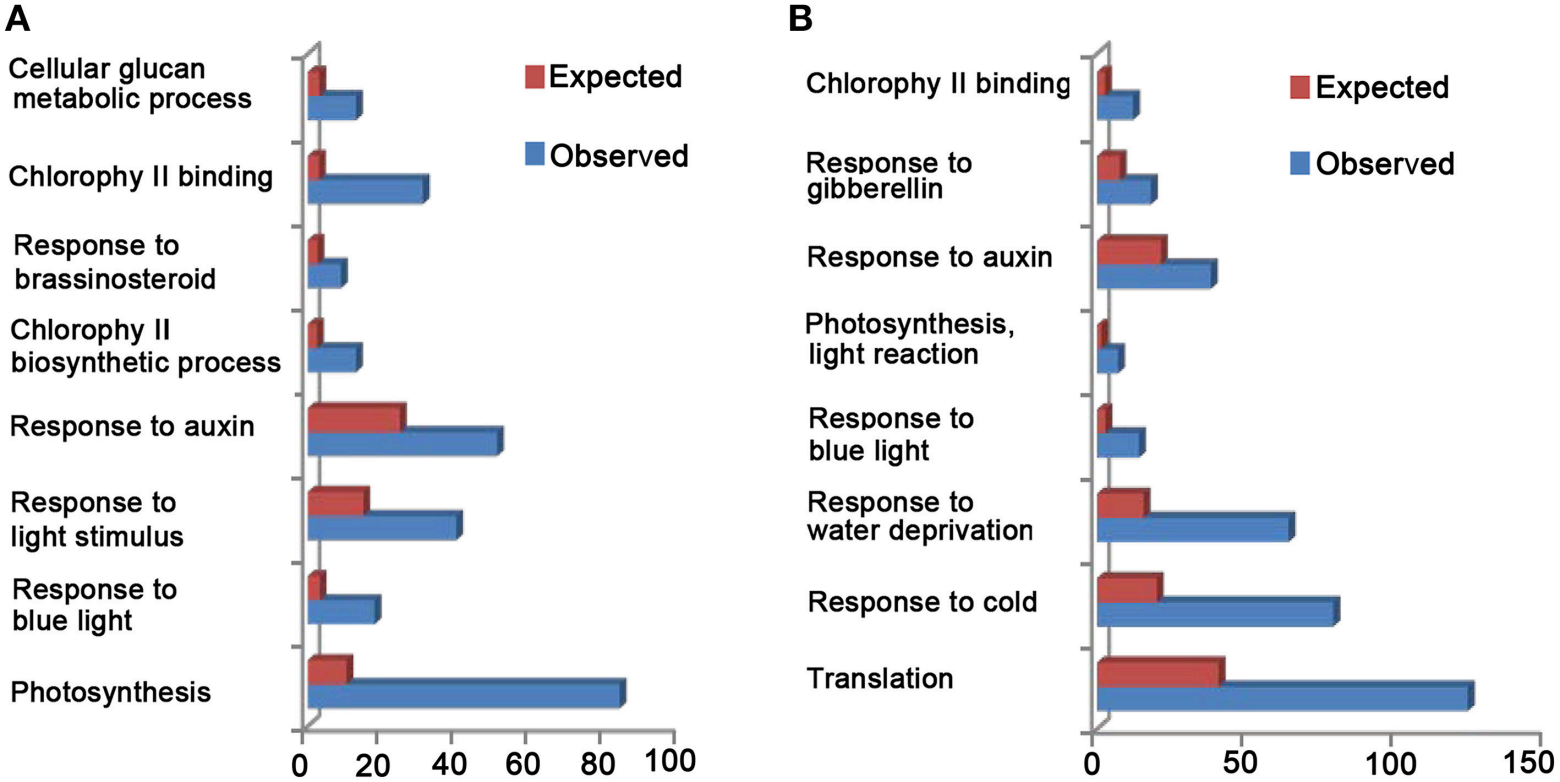
**Gene Ontology (GO) analyses of all the DEGs between WT and *CCT1*, CCT1, and CNT1. (A)** GO enrichment analysis of DEGs between WT and *CCT1*. Expect means the gene number base on all *Arabidopsis* genes. Observed means the gene number base on the DEGs of WT and *CCT1*.**(B)** GO enrichment analysis of DEGs between CCT1 and CNT1. Expect means the gene number base on all *Arabidopsis* genes. Observed means the gene number base on the DEGs of CCT1 and CNT1.

Since CRY is shown to be related to many biological processes, such as “photosynthesis,” “chloroplast organization,” “cell wall organization or biogenesis,” “response to auxin,” “response to GA,” “response to BR,” “response to ABA,” “response to JA,” and “response to SA” (Folta et al., 2003; He et al., 2015), we examined whether CCT1 might be involved in regulating these processes through GO and KEGG analyses, respectively. We found that the DEGs of CCT1 can be classified into 128 GO terms (Table S2), which suggest that CCT1 is associated with several biological processes, such as “photosynthesis,” “response to blue light,” “response to light stimulus,” “response to auxin,” and “response to brassinosteroid” (Figure 2A). KEGG pathway analysis further showed that many DEGs are mapped to “photosynthesis” and “plant hormone signal transduction” pathways (Figure S1A). These results indicated that CCT1 might be involved in mediating CRY1 regulation of phytohormone-responsive gene expression. Since CCT1 and CNT1 co-regulate a large number of genes, which include phytohormone-responsive genes that are regulated by CRY1, we further determined the potential functional relevance of CCT1 and CNT1 through GO and KEGG analyses, respectively. GO analysis revealed that both CCT1 and CNT1 are associated with many biological processes, such as “translation,” “response to cold,” “response to water deprivation,” “response to blue light,” “photosynthesis, light reaction,” “response to auxin,” and “response to gibberellin” (Figure 2B). And KEGG analysis demonstrated that the genes co-regulated by CCT1 and CNT1 mainly participated in “ribosome” pathway (Figure S1B). Taken together, these results indicated that CCT1 alone tends to be involved in mediating CRY1 regulation of photosynthesis, light response, and hormonal regulation similar to CNT1, whereas CCT1 and CNT1 are jointly involved in mediating CRY1 regulation of protein synthesis and stress responses.

### CCT1 and CNT1 mediate CRY1 regulation of GA/BR/auxin-responsive genes

**Figure 3:**
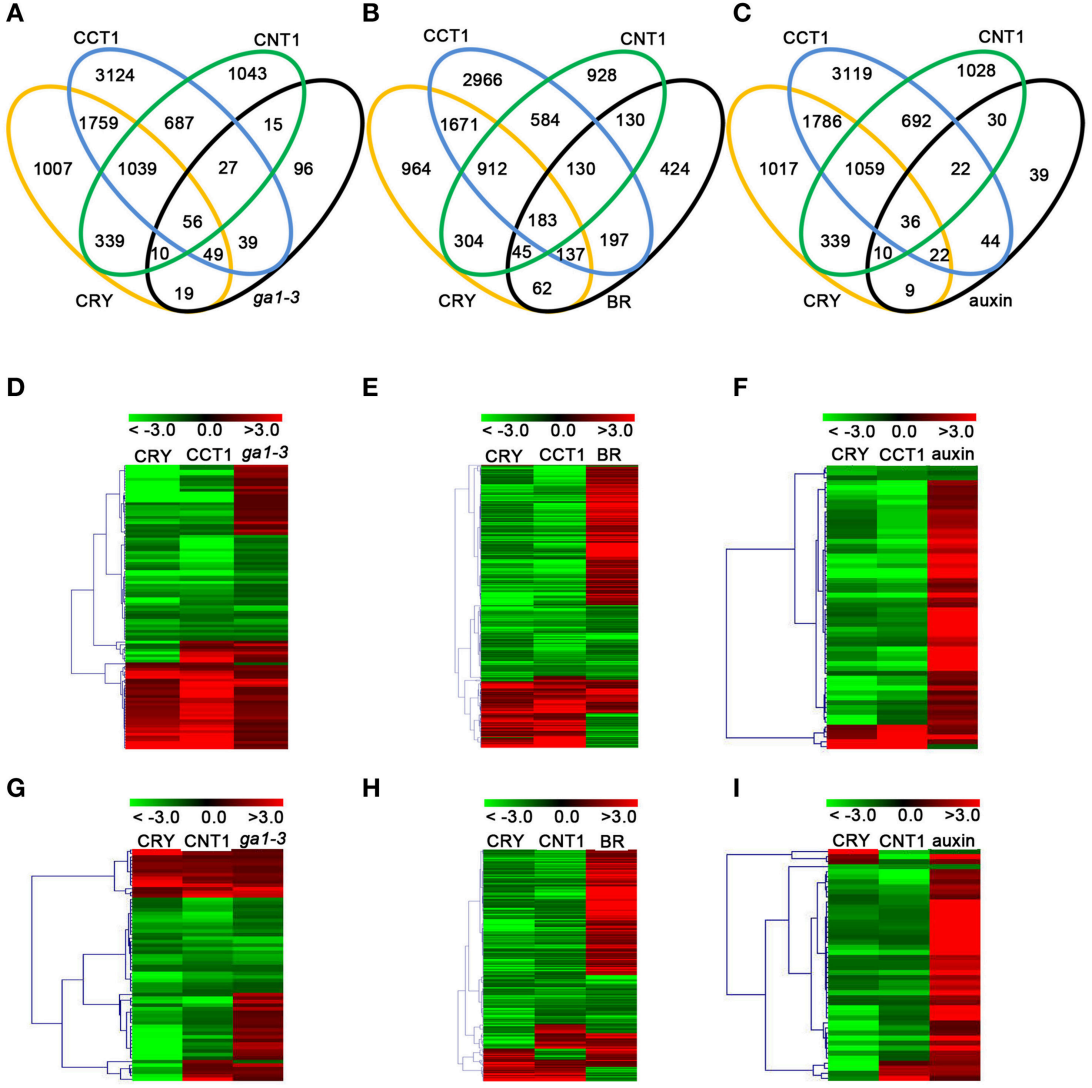
**CRY, CCT1, and CNT1 regulate a large number of GA/BR/auxin-responsive genes. (A–C)** Venn diagram showing the number of unique and common DEGs among CRY, CCT1, CNT1, and GA **(A)**, BR **(B)**, auxin **(C)**. **(D)** Hierarchical clustering analysis of 105 CRY/CCT1/*g*a1-3 overlapping genes as shown in **(A)**. **(E)** Hierarchical clustering analysis of 320 CRY/CCT1/BR overlapping genes as shown in **(B)**. **(F)** Hierarchical clustering analysis of 58 CRY/CCT1/auxin overlapping genes as shown in **(C)**. **(G)** Hierarchical clustering analysis of 66 CRY/CNT1/*ga1-3* overlapping genes as shown in **(A)**. **(H)** Hierarchical clustering analysis of 228 CRY/CNT1/BR overlapping genes as shown in **(B)**. **(I)** Hierarchical clustering analysis of 46 CRY/CNT1/auxin overlapping genes as shown in **(C)**. Red and green colors in the heat-maps represent induced and repressed genes, respectively. Scale bar denotes the log_2_ value of fold change.

With the demonstrations that CCT1 is involved in CRY1 regulation of phytohormone-responsive genes, we further confirmed the relationship among CRY1, CCT1, and CNT1 in regulating phytohormone-responsive genes. We compared the CRY1-, CCT1-, and CNT1-regulated genes with the transcriptomic data relative to GA/BR/auxin reported previously (Nemhauser et al., 2006; Cheminant et al., 2011; Oh et al., 2014), respectively. Of the 311 GA-regulated genes, 134 (43.09%) genes were regulated by CRY, and of these 134 genes, 105 (78.35%) and 66 (49.25%) genes were also regulated by CCT1 and CNT1, respectively, and 56 genes were co-regulated by CCT1 and CNT1 (Figure 3A). Similarly, of the 1308 BR-regulated genes, 427 (32.65%) were regulated by CRY, and of these 427 genes, 320 (74.94%), and 228 (53.40%) genes were also regulated by CCT1 and CNT1, respectively, and 183 genes were co-regulated by CCT1 and CNT1 (Figure 3B). Of the 211 auxin-regulated genes, 77 (47.39%) were regulated by CRY, and of these 77 genes, 58 (75.32%) and 46 (59.74%) genes were also regulated by CCT1 and CNT1, respectively, and 36 genes were co-regulated by CCT1 and CNT1 (Figure 3C). These results confirmed that both CCT1 and CNT1 participate in CRY1 regulation of phytohormone-responsive genes.

Heat-map analyses showed that most of the 105 genes co-regulated by CRY, CCT1 and GA were regulated by CRY and CCT1 in the same direction, and that about one third of these genes were up-regulated by GA, but down-regulated by CRY and CCT1 (Figure 3D). Similarly, most of the 320 genes co-regulated by CRY, CCT1, and BR (Figure 3E), and 58 genes co-regulated by CRY, CCT1, and auxin (Figure 3F), were also regulated by CRY and CCT1 in the same direction, respectively, and a number of these genes were up-regulated by BR/auxin, but down-regulated by CRY and CCT1. Moreover, 66 genes co-regulated by CRY, CNT1, and GA (Figure 3G), and 228 genes co-regulated by CRY, CNT1, and BR (Figure 3H), and 46 genes co-regulated by CRY, CNT1, and auxin (Figure 3I), were regulated by CRY and CNT1 in the same direction, and part of these genes were up-regulated by GA/BR/auxin, but down-regulated by CRY and CNT1. These results suggest that the antagonistic regulation of hypocotyl elongation by CRY1 and GA/BR/auxin is likely mediated through CCT1- and CNT1-mediated CRY1 inhibition of genes responsive to GA/BR/auxin.

### CCT1 and CNT1 specifically regulate yet co-regulate some of the GA/BR/auxin-responsive genes

**Figure 4:**
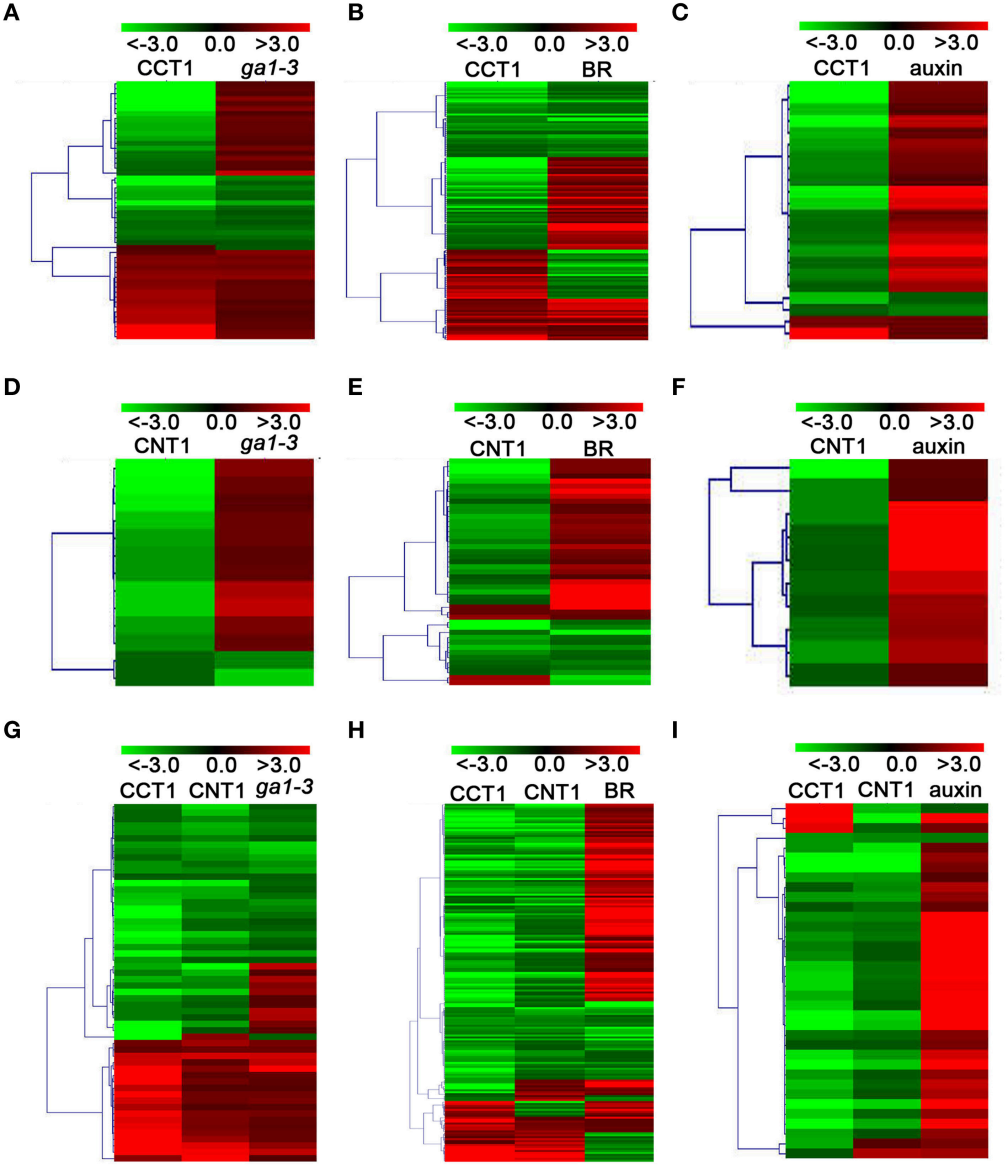
**CCT1 and CNT1 specifically regulate yet co-regulate a large number of the GA/BR/auxin-responsive genes. (A)** Hierarchical clustering analysis of 49 CRY/CCT1/*ga1-3* overlapping genes as shown in (Figure 3A). **(B)** Hierarchical clustering analysis of 137 CRY/CCT1/BR overlapping genes as shown in (Figure 3B). **(C)** Hierarchical clustering analysis of 22 CRY/CCT1/BR overlapping genes as shown in (Figure 3C). **(D)** Hierarchical clustering analysis of 10 CRY/CNT1/*ga1-3* overlapping genes as shown in (Figure 3A). **(E)** Hierarchical clustering analysis of 45 CRY/CNT1/BR overlapping genes as shown in (Figure 3B). **(F)** Hierarchical clustering analysis of 10 CRY/CNT1/auxin overlapping genes as shown in (Figure 3C). **(G)** Hierarchical clustering analysis of 56 CRY/CCT1/CNT1/*ga1-3* overlapping genes as shown in (Figure 3A). **(H)** Hierarchical clustering analysis of 183 CRY/CCT1/CNT1/BR overlapping genes as shown in (Figure 3B). **(I)** Hierarchical clustering analysis of 36 CRY/CCT1/CNT1/auxin overlapping genes as shown in (Figure 3C). Red and green colors in the heat-maps represent induced and repressed genes, respectively. Scale bar denotes the log_2_ value of fold change.

To further verify the potential relationship between CCT1 and CNT1 in regulating phytohormone-responsive genes, we analyzed the CCT1- and CNT1-regulated genes that are regulated by CRY in details. We found that, of the 134 GA- and CRY-co-regulated genes, 49 and 10 genes were specifically regulated by CCT1 and CNT1, respectively, and 56 genes were co-regulated by CCT1 and CNT1. Similarly, of the 427 BR- and CRY-co-regulated genes, 137 and 45 genes were specifically regulated by CCT1 and CNT1, respectively, and 183 genes were co-regulated by CCT1 and CNT1. Of the 77 auxin- and CRY-co-regulated genes, 22 and 10 genes were specifically regulated by CCT1 and CNT1, respectively, and 36 genes were co-regulated by CCT1 and CNT1 (Figures 3A–C). The aforementioned genes were indicated by hierarchical clustering analyses, respectively (Figure 4), and heat-map graphs showed that more than half of the GA/BR/auxin up-regulated genes were down**-**regulated by CCT1 and CNT1, respectively (Figures 4A–F), or both of them (Figures 4G–I). Since CRY1 and GA/BR/auxin act antagonistically to regulate hypocotyl elongation, we further analyzed the genes that are up-regulated by GA/BR/auxin but down-regulated by CRY, CCT1, and CNT1(Table S3), and found that many of these genes have been reported to regulate hypocotyl elongation, including *XTHs, SAURs, IAAs, PREs, BBXs*, and *PARs* (Kim et al., 1996; Zhang et al., 2009; Sasidharan et al., 2010; Hao et al., 2012; Spartz et al., 2012; Gangappa and Botto, 2014). Taken together, these results indicate that CCT1 and CNT1 may mediate CRY1 signaling by specifically regulating yet co-regulating some of the phytohormone-responsive genes.

### Validation of differentially expressed genes by RT-qPCR

**Figure 5:**
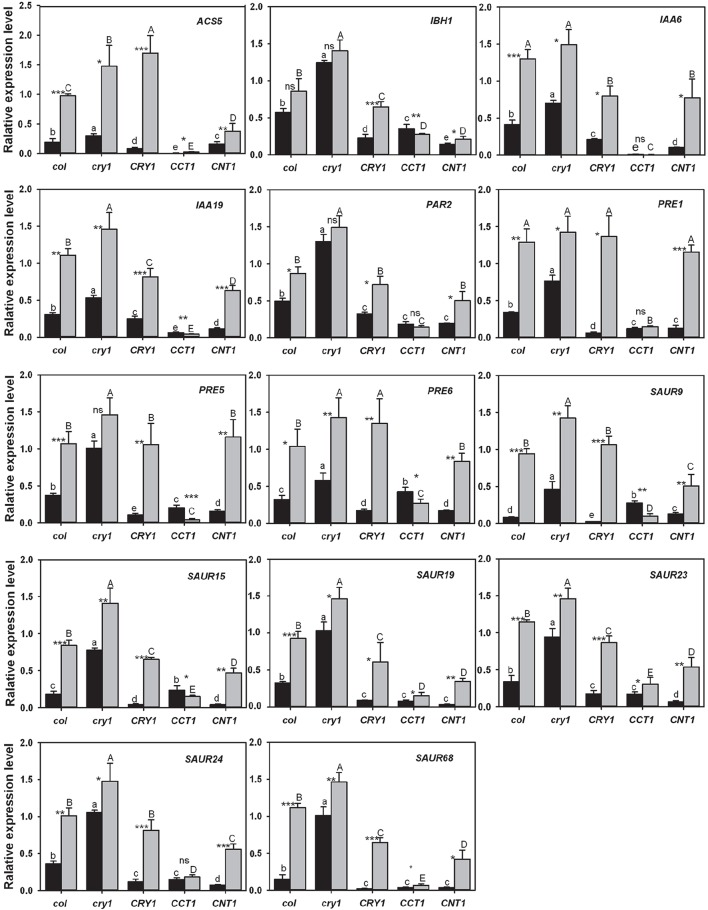
**qRT–PCR analysis showing the effects of blue light, CRY1, CCT1, and CNT1 on GA/BR/ auxin-responsive gene expression**. qRT–PCR analysis showing the effects of blue light, CRY1, CCT1, and CNT1 on the genes that are relative to hypocotyl elongation and are down-regulated by CRY, CCT1, or CNT1, but up-regulated by phytohormones. Data are mean ± SD (*n* = 3). Asterisks denote significant differences in the gene expression level between the two compared conditions of blue light (black columns) and darkness (gray columns) (*Significant at *P* < 0.05; **Significant at *P* < 0.01; ***Significant at *P* < 0.001, and ns denotes no significant differences at *P*>0.05) as determined by Student's t test. The lowercase letters (a–e) indicate significant differences among means for the gene expression level of five genotypes of blue-light-grown seedlings, as determined by LSD (least significant difference) at a significance level of 0.05. The uppercase letters (A–E) indicate significant differences among means for the gene expression level of five genotypes seedlings grown in darkness, as determined by LSD (least significant difference) at a significance level of 0.05.

To further confirm the gene expression patterns observed from the transcriptomic analyses, we performed reverse transcription-quantitative PCR (RT-qPCR) analyses with the genes that are relative to hypocotyl elongation and down-regulated by CRY, CCT1, and CNT1 (Table S4), but up-regulated by GA/BR/auxin. We firstly determined whether these genes might be regulated by CRY1-mediated blue light signaling. Indeed, almost all of these genes expressed at significantly higher levels in WT seedlings grown in the dark than in those exposed to blue light (Figure 5). Moreover, upon blue light irradiation, these genes expressed at much higher levels in *cry1* mutant than in WT, whereas they expressed at considerably lower levels in *CRY1*-overexpressing (*CRY1-ovx*) seedlings than in WT seedlings (Figure 5). We further determined the role for CCT1 or CNT1 in mediating CRY1 regulation of these genes through RT-qPCR, and found that the identified genes specifically regulated by CCT1 indeed expressed at a lower level in *CCT1* than in *CNT1* seedlings exposed to blue light, such as *ACS5*. Similarly, the identified CNT1-specific genes expressed at a lower level in *CNT1* than in *CCT1* seedlings exposed to blue light, such as *SAUR15* (Figure 5). Furthermore, all of the identified CCT1- and CNT1-co-regulated genes are indeed down-regulated by CCT1 and CNT1, such as *SAUR19* (Figure 5). These results indicated that our RNA-Seq analyses are reliable, and therefore further support the involvement of CCT1 and CNT1 in mediating CRY1 regulation of phytohormone-responsive gene expression.

### Both CCT1 and CNT1 are involved in mediating CRY1 reduction of the seedlings sensitivity to GA

**Figure 6:**
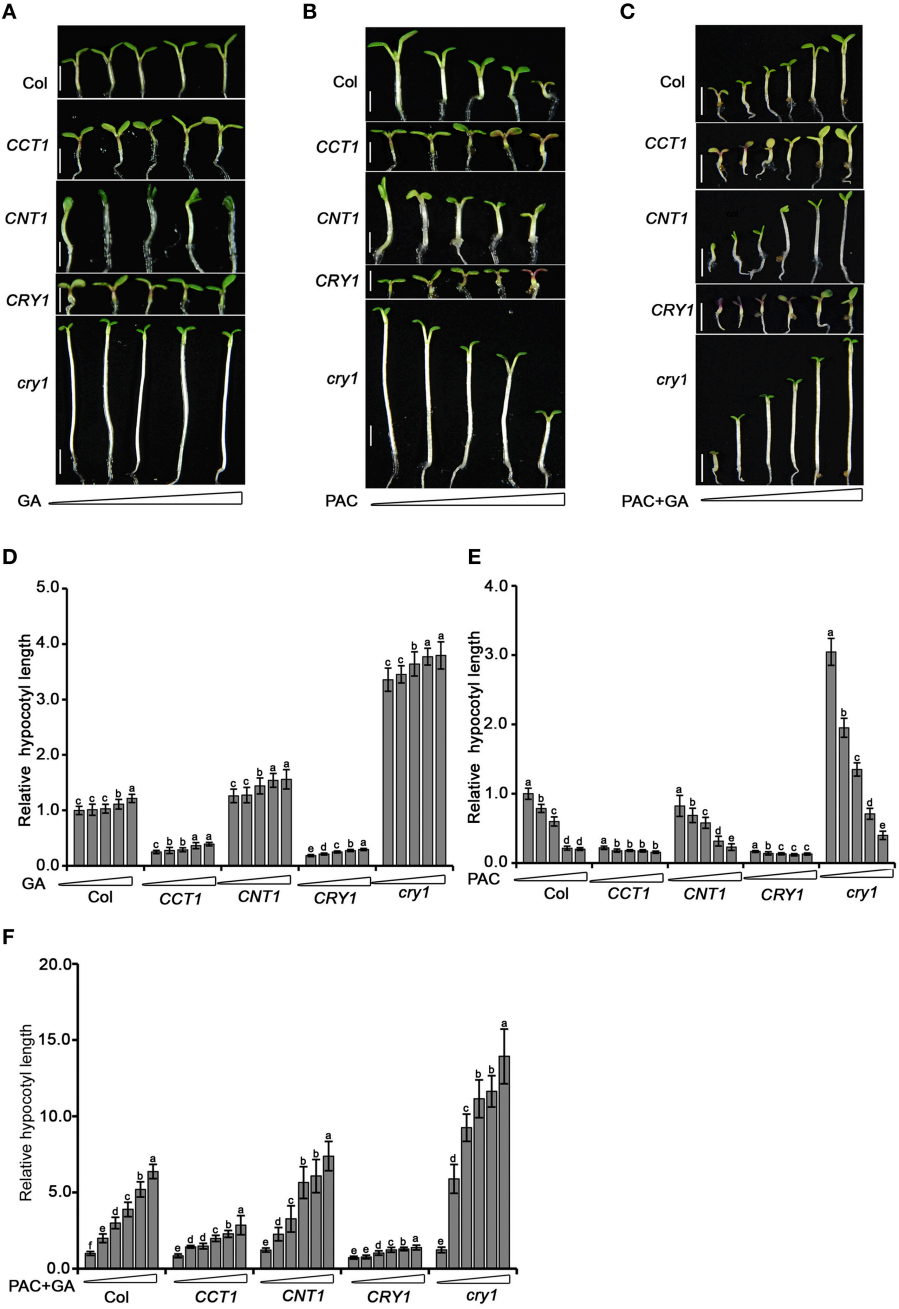
**Hypocotyl elongation analysis of WT, *cry1* mutant, *CRY1-ovx*, *CCT1*, and *CNT1* in response to GA or PAC. (A)** Five-day-old *Arabidopsis* WT, *cry1* mutant, *CRY1-ovx, CCT1*, and *CNT1* seedlings grown on half-strength MS plates supplemented gradient concentrations (0, 0.05, 0.2, 0.5, 1 μM) of GA under 30 μmol/m^2^/s blue light. **(B)** Five-day-old *Arabidopsis* WT, *cry1* mutant, *CRY1-ovx, CCT1*, and *CNT1* seedlings grown on half-strength MS plates supplemented with gradient concentrations of PAC (0, 0.025, 0.05, 0.2, 0.5 μM) under 30 μmol/m^2^/s blue light. Means ± SD were obtained from 30 independent plants. **(C)** Six-day-old *Arabidopsis* WT, *cry1* mutant, *CRY1-ovx, CCT1*, and *CNT1* seedlings grown on half-strength MS plates supplemented with 1 μM PAC plus gradient concentrations of GA (0, 0.5, 1.0, 2.5, 5.0, 10.0 μM) under 30 μmol /m^2^/s blue light. **(D)** Hypocotyl length measurement of blue-light-grown seedlings treated with increasing amounts of GA. The concentrations of GA used are 0, 0.05, 0.2, 0.5, and 1 μM (from left to right). **(E)** Hypocotyl length measurement of blue-light-grown seedlings treated with increasing amounts of PAC. The concentrations of PAC used are 0, 0.025, 0.05, 0.2, and 0.5 μM (from left to right). **(F)** Hypocotyl length measurement of blue-light-grown seedlings treated with 1 μM PAC plus gradient concentrations of GA. The concentrations of GA used are 0, 0.5, 1.0, 2.5, 5.0, 10.0 μM (from left to right). Seedlings in **(D–F)** were grown as described in **(A–C)**, respectively. In **(D–F)**, hypocotyl length of untreated wild-type seedlings is set to 100%. Scale bars in **(A–C)** represent 2.5 mM. The letters (a–f) in **(D–F)** indicate significant differences among means for the hypocotyl lengths of seedlings treated with the gradient concentration of GA, PAC, PAC plus GA, as determined by LSD (least significant difference) at a significance level of 0.05.

With the demonstration that CCT1 and CNT1 are involved in mediating CRY1 regulation of phytohormone-responsive gene expression, we further explored whether CCT1 and CNT1 are physiologically involved in the regulation of phytohormone response by CRY1. To do this, we tested the hypocotyl elongation response of seedlings to GA and its biosynthesis inhibitor, paclobutrazol (PAC), which are shown to promote and inhibit hypocotyl elongation, respectively (Feng et al., 2008). We found that *cry1* mutant shows a long hypocotyl phenotype upon blue light irradiation, but responded only weakly to the increased concentrations of GA compared to WT. However, *cry1* mutant showed significantly more sensitive responsiveness to PAC than WT (Figures 6A,B,D,E), indicating that the endogenous GA levels are likely higher in *cry1* mutant than in WT (Zhao et al., 2007). In an attempt to minimize the potential GA content difference in WT, *cry1* mutant, *CRY1-ovx, CCT1*, and *CNT1* seedlings and obtain more dramatic difference in the responsiveness of these genotypes of plants to GA, we treated these plants firstly with PAC and then with GA, respectively. Indeed, *cry1* mutant seedlings exhibited significantly greater responsiveness to GA than WT (Figures 6C,F), confirming the role for CRY1 in repressing GA response (Zhao et al., 2007). However, either *CRY1-ovx* or *CCT1* seedlings hardly responded to GA in either the presence or absence of PAC (Figures 6A–F). Similarly, *CNT1* seedlings, which are in the *cry1* mutant background, displayed significantly more reduced responsiveness to GA (Figures 6A–F). Taken together, these results indicate that CCT1 and CNT1 are involved in CRY1 inhibition of GA response.

### CCT1 mediates CRY1 inhibition of GA-promoted degradation of HY5 protein

**Figure 7:**
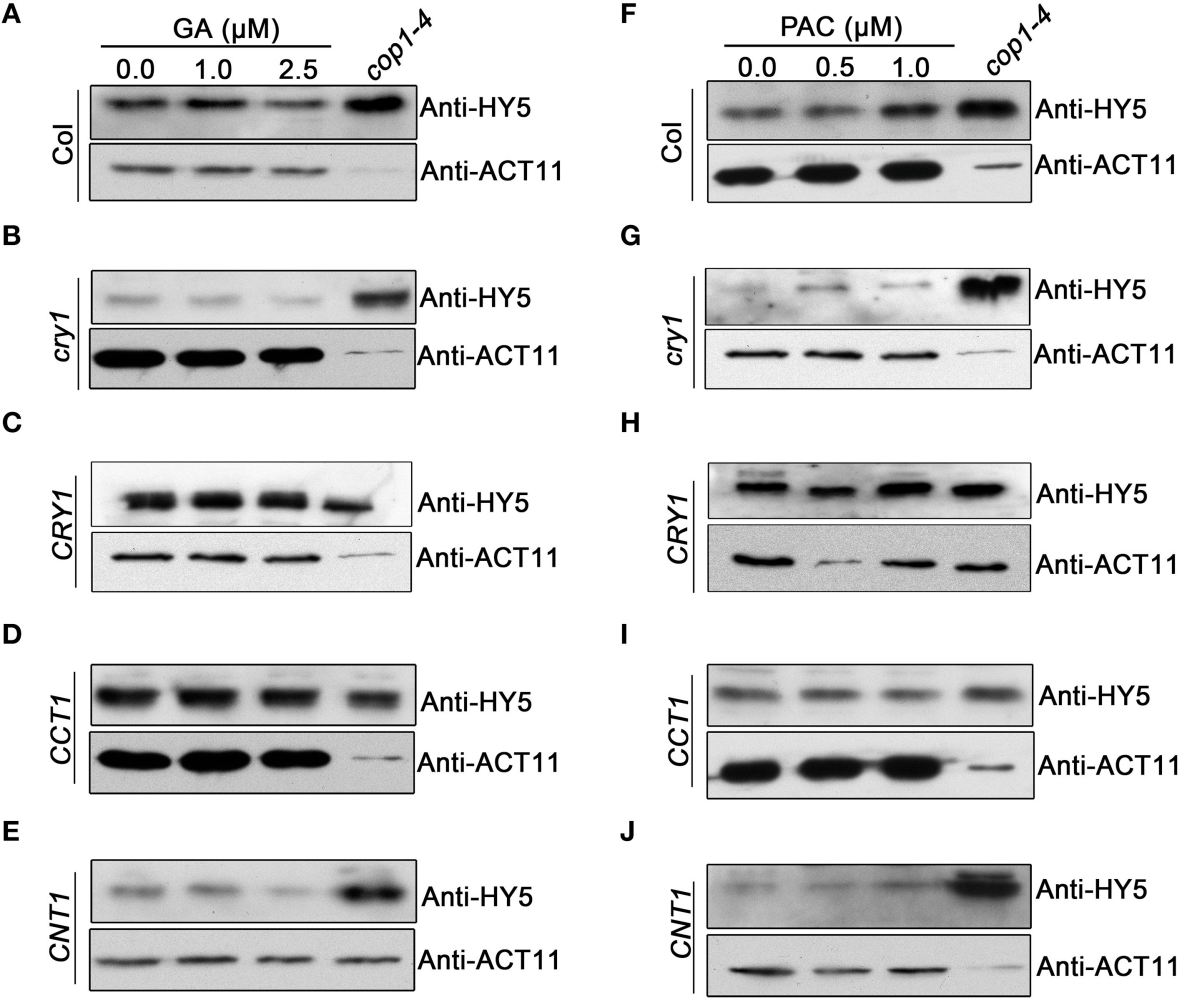
**Western blotting assay showing the effects of CRY1, CCT1, and CNT1 on GA or PAC regulation of HY5 protein level**. **(A–E)** HY5 protein levels in blue light-grown WT **(A)**, *cry1* mutant **(B)**, *CRY1* **(C)**, *CCT1* **(D)** and *CNT1* **(E)** plants, as determined by Western blotting using anti-HY5 antibody. Seedlings were grown for 4 days under blue light on half-strength MS media with agar and gradient GA (0, 1, 2.5 μM) concentrations. **(F–J)** HY5 protein levels in blue light-grown WT **(F)**, *cry1* mutant **(G)**, *CRY1* **(H)**, *CCT1* **(I)** and *CNT1* **(J)** plants, as determined by Western blotting using anti-HY5 antibody. Seedlings were grown for 4 days under blue light on half-strength MS media with agar and gradient PAC (0, 0.5, 1 μM) concentrations. Anti-HY5 antibody detection in *cop1-4* acted as a positive control. Endogenous actin determined by anti-ACT11 was used as a loading control.

HY5 is known to be an important positive regulator of photomorphogenesis, whose protein stability is promoted by light (Osterlund et al., 2000), but reduced by GA (Alabadi et al., 2008). To explore whether CRY1 is involved in regulating GA-promoted degradation of HY5, we performed Western blotting assay using an anti-HY5 antibody to analyze the potential changes of HY5 protein in WT, *cry1* mutant, and *CRY-ovx* seedlings in response to GA and PAC treatments, respectively. We observed that the HY5 protein level in *cry1* mutant was very low and reduced much faster compared to WT upon GA application (Figures 7A,B). In contrast, upon PAC application, HY5 accumulates more slowly in *cry1* mutant than in WT (Figures 7F,G). Interestingly, HY5 protein level in *CRY1-ovx* seedlings is basically not affected by GA treatments (Figure 7C). These results indicated that CRY1 is involved in repressing GA-promoted degradation of HY5 protein. We further analyzed HY5 protein levels in *CCT1* and *CNT1* seedlings treated with GA and PAC, respectively. The results indicate that the dynamic changes of HY5 protein levels in *CNT1* seedlings, which are in *cry1* mutant background, are basically similar to *cry1* mutant (Figures 7E,J), indicating that CNT1 is not likely involved in CRY1 inhibition of GA-promoted HY5 protein degradation. However, GA reduction of HY5 protein levels is inhibited in *CCT1* seedlings (Figure 7D), indicating that CCT1 may mediate CRY1 repression of GA-induced HY5 degradation.

## Discussion

### Both CCT1 and CNT1 regulate many target genes of CRY1 in genome-wide

The C terminus of CRY1 was shown to mediate CRY1 signaling over 14 years ago (Yang et al., 2000, 2001). Most recently, it has been demonstrated that the N terminus of CRY1 is also involved in CRY1 signaling, which is independent on its C terminus (He et al., 2015). In the present study, we obtained 6780 CCT1-regulated genes through RNA-Seq and compared these genes with those shown to be regulated by CRY and CNT1 previously (He et al., 2015). The results demonstrate the involvement of CCT1 in the regulation of gene expression at the transcriptomic level, which facilitated us to obtain the global “digital” transcriptional information of CCT1. We found that CCT1 influences 2903 (67.85%) of the CRY-regulated genes (Figure 1A), and that CRY-, CCT1-, and CNT1-co-regulated genes are modulated in the same direction (Figure 1B), indicating a close correlation of CCT1 and CNT1 with CRY1 in their regulation of gene expression. Although a previous study analyzed the CCT1 and CCT2 global genome expression profiles using an *Arabidopsis* cDNA microarray (Wang et al., 2001), its aim was to prove whether transgenic *CCT1* and *CCT2* seedlings share a similar global genome expression profile to *cop1* mutant. In this study, our RNA-Seq analysis not only generated much more target genes than cDNA microarray assay, but revealed the potential common and specific gene targets regulated by CCT1 and CNT1 at the transcriptomic level, and all these genes are also regulated by CRY.

### Both CCT1 and CNT1 are involved in mediating CRY1 regulation of phytohormone-responsive genes in genome-wide

GO and KEGG analyses suggest that CCT1 participate in several biology processes, similar to CRY (He et al., 2015), of which the process of “response to many hormones” is one of the most enriched (Figure 2A). Most of overlapping genes regulated by CRY and GA/BR/auxin also were regulated by CCT1 and CNT1 (Figure 3). However, some of the genes regulated by GA/BR/auxin are specifically affected by CCT1 or CNT1 (Figure 4). RT-qPCR assay further confirmed that all the genes that respond to GA/BR/auxin and are related to the regulation of hypocotyl elongation are indeed down-regulated by CRY1, which is likely mediated by CCT1 or CNT1 only or them both (Figure 5). Physiological studies demonstrate that both CCT1 and CNT1 are involved in mediating CRY1 inhibition of GA-enhanced seedlings hypocotyl elongation (Figures 6A–F). Protein expression studies suggested that CCT1 may mediate CRY1 repression of GA-induced HY5 degradation, but not CNT1 (Figures 7A–E). These results, in conjunction with the global genome expression profiles of CRY (He et al., 2015) and CCT1 (this study), indicate that CCT1 and CNT1 may be involved in CRY1 regulation of GA and likely other phytohormones response.

### Possible mechanisms of CCT1 and CNT1 in mediating CRY1 regulation of phytohormone-responsive genes

Our transcriptome analysis results show that CCT1/CNT1 specifically regulate many GA/BR/auxin-responsive genes that are regulated by CRY (Figures 3, 4), indicating that CCT1 and CNT1 may adopt different mechanism to mediate CRY1 regulation of phytohormone-responsive genes. Since CCT1 and CCT2 mediate CRY1 and CRY2 signaling through direct interaction with COP1 (Wang et al., 2001; Yang et al., 2001), and CRY1 inhibition of GA-promoted HY5 degradation is mediated by CCT1 (Figure 7), we propose that CCT1-mediated CRY1 regulation of GA response may be mediated through CCT1 regulation of COP1. To test this prediction, we performed RNA-Seq analysis using *cop1-4* mutant (Table S5) and obtained 5774 COP1-regulated genes (Table S6). Vern diagram showed that CRY and CCT1 regulate a large number of overlapping genes with COP1 in an opposite direction, which is consistent with their opposite role in the regulation of hypocotyl elongation (Figures S2A,B). Given that COP1 is shown to participate in many phytohormones signaling, including GA/BR/auxin (Alabadi and Blazquez, 2008; Luo et al., 2010; Sassi et al., 2012), these results, in conjunction with the previous microarray data obtained from *cop1* mutant (Wang et al., 2001), suggest that CCT1 may regulate phytohormone-responsive genes mainly through regulation of COP1. We also found that CRY and CNT1 regulate a large number of overlapping genes with COP1 in an opposite direction (Figures S3A,B), some of which are GA/BR/auxin-responsive genes (Figures S4A–I). Since CNT1 does not interact with COP1 (Yang et al., 2001), and the enhancement of blue light responsiveness by CNT1 is not mediated through promotion of HY5 accumulation (He et al., 2015), we postulate that CNT1 regulation of phytohormone genes, as well as GA response, may not involve its direct regulation of COP1. However, the downstream components of COP1, including its direct substrates and potentially the components involved in phytohormone signaling, can be directly or indirectly regulated by CNT1, which will be worth exploring in future studies.

It is interesting to note that CCT1 and COP1 do not always regulate the same phytohormone-responsive genes (Figures S4A–C), suggesting that CCT1-mediated CRY1 signaling may not exclusively proceed through COP1. It is shown that light and GA signaling is integrated through direct interaction of DELLA proteins, key negative regulators in GA signaling pathway (Achard et al., 2007), with PIF3 and PIF4, pivotal negative transcriptional factors of photomorphogenesis (Leivar et al., 2008), resulting in inhibition of their DNA-binding activities (de Lucas et al., 2008; Feng et al., 2008). CCT1 may regulate these key factors of light and phytohormone signaling to mediate CRY1 regulation of phytohormone response. In view of the demonstration that CNT2 mediates the interaction of CRY2 with CIBs, bHLH transcription factors, to promote the binding to the promoter of *FT* to regulate flowering, it will also be worth exploring whether CRY1 interacts with the components in GA/BR/auxin signaling pathways to regulate phytohormone-responsive gene expression and physiological response, through CCT1 or CNT1. Taken together, this study provides clue to further exploring the molecular mechanisms by which CCT1 and CNT1 mediate CRY1 signaling, which may involve regulation of phytohormone-responsive gene expression.

## Author contributions

WW and HY designed the project; WW, HL, and ZM performed the experiments; WW analyzed the data; WW and HY wrote the manuscript; all authors reviewed and edited the manuscript.

### Conflict of interest statement

The authors declare that the research was conducted in the absence of any commercial or financial relationships that could be construed as a potential conflict of interest.
