# Supplementary material for: Transcriptome Analyses Reveal the Involvement of Both C and N Termini of Cryptochrome 1 in Its Regulation of Phytohormone-Responsive Gene Expression in Arabidopsis
Source: Front Plant Sci. 2016 Mar 14;7:294. doi: 10.3389/fpls.2016.00294 (PMC4789503; doi:10.3389/fpls.2016.00294)
Supplement: Supplementary file 1 [file Presentation1.PDF]

Frontiers in Plant Science

***Supplementary Material*:**

Transcriptome analyses reveal the involvement of both C and N termini of cryptochrome 1 in its regulation of phytohormone-responsive gene expression in *Arabidopsis*

Wen-Xiu Wang[1], Hong-Li Lian[1], Li-Da Zhang[1], Zhi-Lei Mao[2], Xiao-Ming Li[2], Feng Xu[1], Ling Li[1] and Hong-Quan Yang[2]*

*Correspondence author.

Hong-Quan Yang, *E-mail address*: hongquanyang@fudan.edu.cn

Figure S1. KEGG enrichment analyses of all the DEGs between WT and *CCT1*, CCT1 and CNT1

(A) KEGG analysis of DEGs between WT and *CCT1*.

(B) KEGG analysis of DEGs between CCT1 and CNT1.

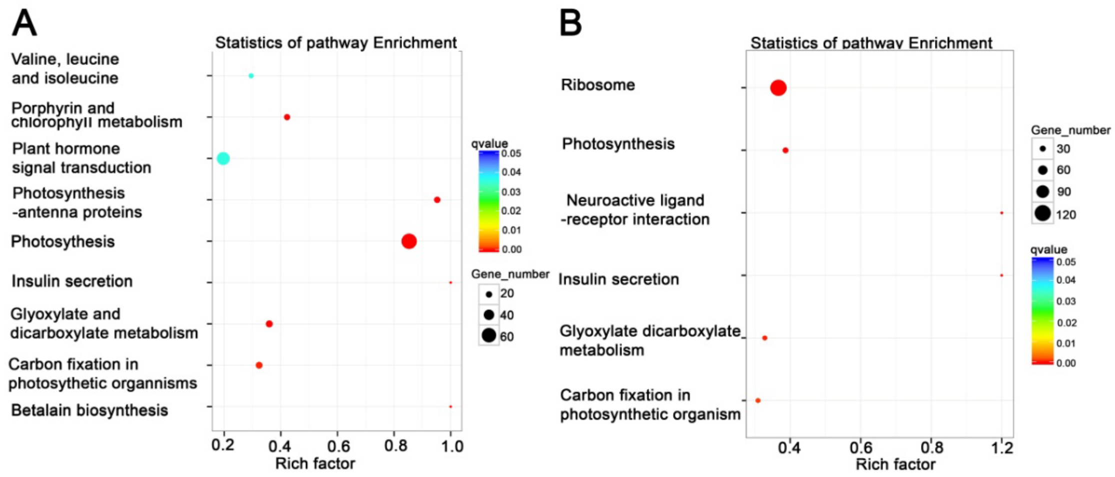

Figure S2. CRY, CCT1 and COP1 regulate a large number of overlapping genes in an opposite direction.

(A) Illustration of the overlapping genes between CRY, CCT1 and COP1.

(B) Hierarchical clustering of the overlapping genes as shown in **(A)**.

Red and green colors in the heat-maps represent induced and repressed genes,respectively. Scalebar denotes the $\log_2$ value of fold change.

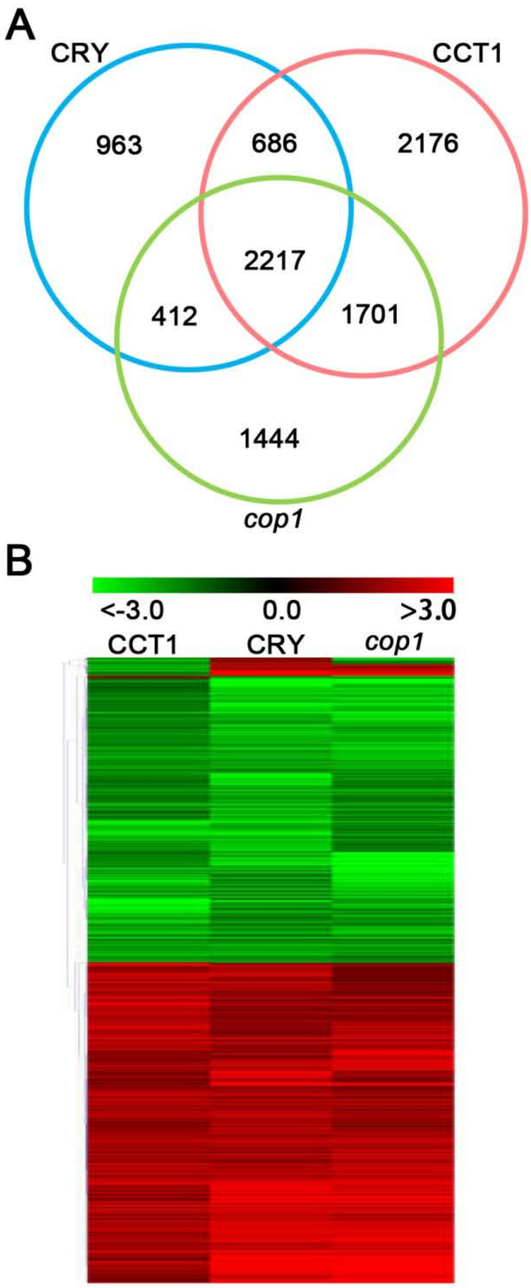

Figure S3. CRY, CNT1 and COP1 regulate a large number of overlapping genes in an opposite direction.

(A) Illustration of the overlapping genes between CRY, CNT1 and COP1.

(B) Hierarchical clustering of the overlapping genes as shown in **(A)**.

Red and green colors in the heat-maps represent induced and repressed genes, respectively. Scalebar denotes the $\log_2$ value of fold change.

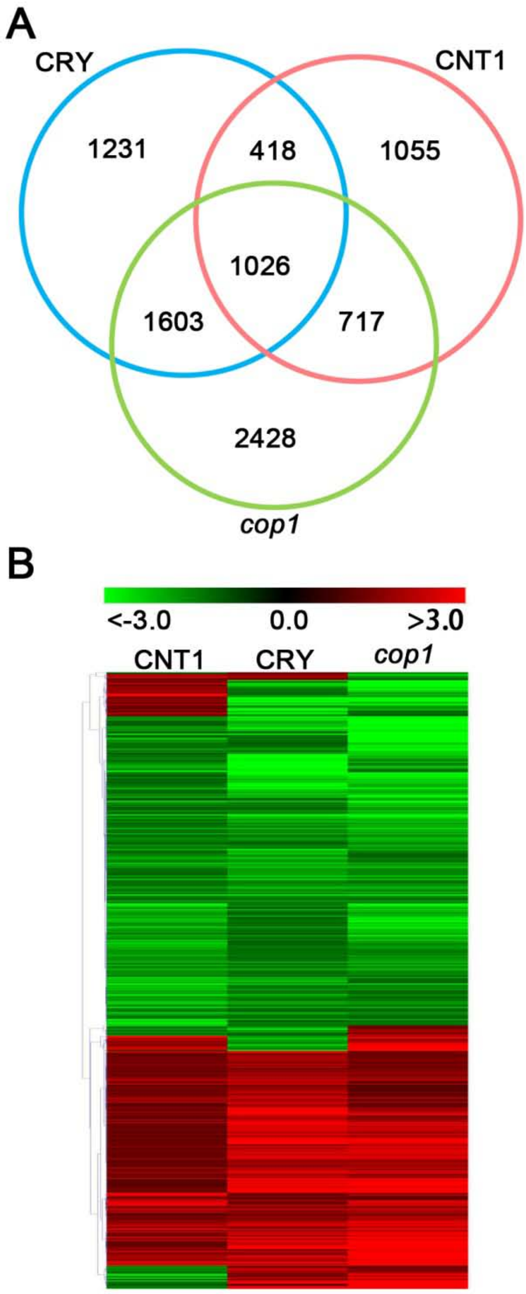

Figure S4. CCT1, CNT1 and COP1 regulate a large number of overlapping genes together with GA/BR/auxin

(A-C) Venn diagram showing the number of unique and common DEGs among CCT1, CNT1, COP1, and GA **(A)**, BR **(B)**, auxin **(C)**.

(D) Hierarchical clustering analysis of 84 COP1/CCT1/*ga1-3* overlapping genes as shown in **(A)**.

(E) Hierarchical clustering analysis of 266 COP1/CCT1/BR overlapping genes as shown in **(B)**.

(F) Hierarchical clustering analysis of 45 COP1/CCT1/auxin overlapping genes as shown in **(C)**.

(G) Hierarchical clustering analysis of 46 COP1/CNT1/ *ga1-3* overlapping genes as shown in **(A)**.

(H) Hierarchical clustering analysis of 165 COP1/CNT1/BR overlapping genes as shown in **(B)**.

(I) Hierarchical clustering analysis of 31 COP1/CNT1/auxin overlapping genes as shown in **(C)**.

Red and green colors in the heat-maps represent induced and repressed genes, respectively. Scalebar denotes the $\log_2$ value of fold change.

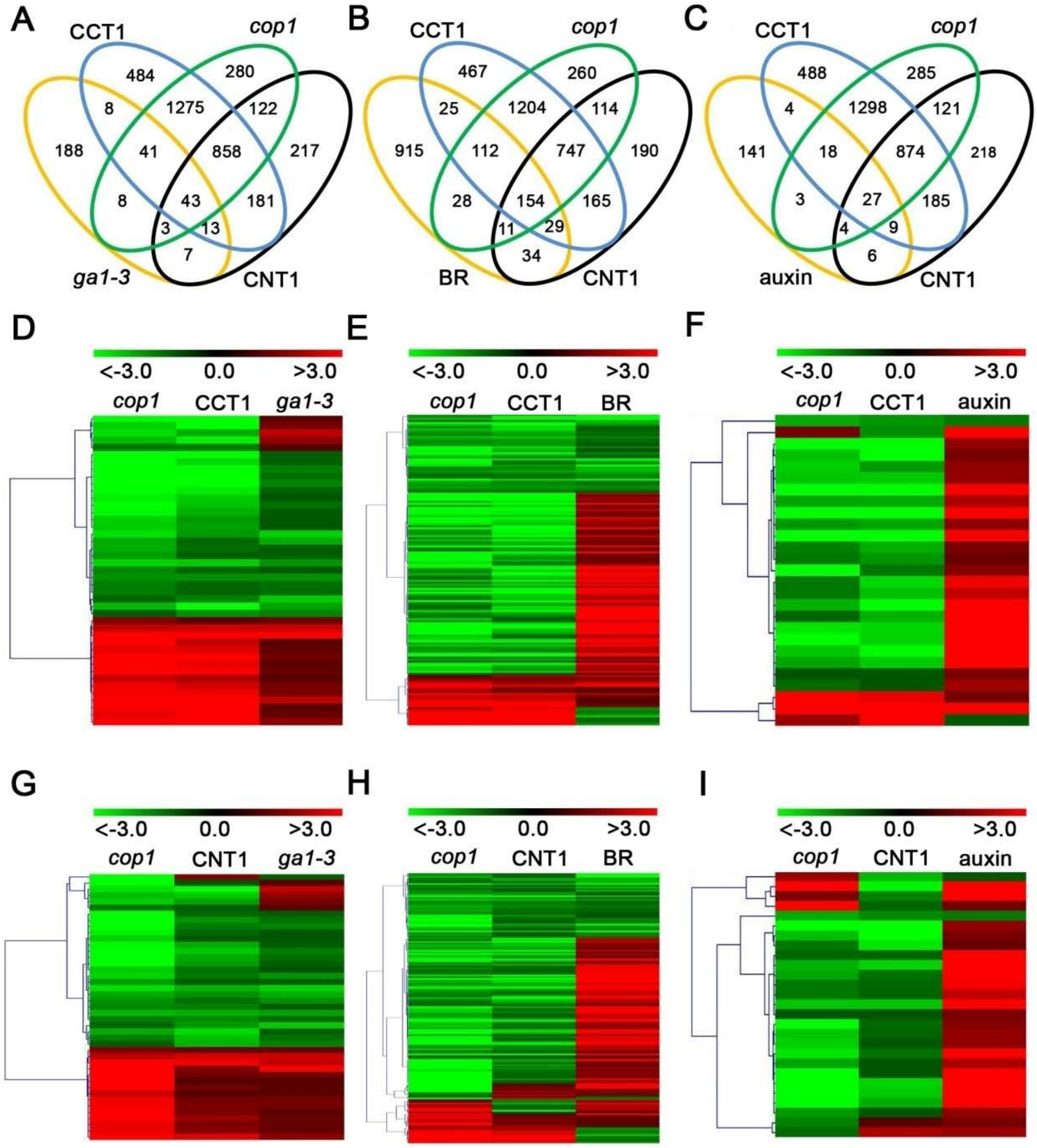

A
CCT1
cop1
ga1-3
CNT1
484
280
8
1275
122
188
41
858
217
8
43
181
3
13
7
B
CCT1
cop1
BR
CNT1
467
260
25
1204
114
915
112
747
190
28
154
165
11
29
34
C
CCT1
cop1
auxin
CNT1
488
285
4
1298
121
141
18
874
218
3
27
185
4
9
6
D
<-3.0
0.0
>3.0
cop1
CCT1
ga1-3
E
<-3.0
0.0
>3.0
cop1
CCT1
BR
F
<-3.0
0.0
>3.0
cop1
CCT1
auxin
G
<-3.0
0.0
>3.0
cop1
CNT1
ga1-3
H
<-3.0
0.0
>3.0
cop1
CNT1
BR
I
<-3.0
0.0
>3.0
cop1
CNT1
auxin

Table S6. Summary of RNA-Seq data from WT and *cop1*

Map to gene	cop1_D1		cop1_D2		WT_D1		WT_D2	
	Reads number	percentage	Reads number	percentage	Reads number	percentage	Reads number	percentage
clean reads	38272295	100%	29719627	100%	25743501	100%	22651750	100%
Total mapped reads	34588576	90.37%	27030527	90.95%	23312549	90.56%	20382779	89.98%
Unique_match	31816700	83.13%	24862727	83.66%	21670195	84.18%	18921019	83.53%
Perfect match	32682769	85.40%	25564559	86.02%	21919460	85.15%	19150500	84.54%
