# Supplementary material for: Transcriptome Analyses Reveal the Involvement of Both C and N Termini of Cryptochrome 1 in Its Regulation of Phytohormone-Responsive Gene Expression in Arabidopsis
Source: Front Plant Sci. 2016 Mar 14;7:294. doi: 10.3389/fpls.2016.00294 (PMC4789503; doi:10.3389/fpls.2016.00294)
Supplement: Supplementary file 5 [file DataSheet4.docx]

**Table S4 Primers used for qPCR analysis.**

| ACT2-F | AAGCTGGGGTTTTATGAATGG |  |
| --- | --- | --- |
| ACT2-R | TTGTCACACACAAGTGCATCAT |  |
| ACS5-R | GAAGGAAATAAAGAAGAAGAAAAAACC |  |
| ACS5-F | GATCGTGTACCTGATGAACGATG |  |
| *IAA19 -F* | GGTGACAACTGCGAATACGTTACCA |  |
| *IAA19-R* | CCCGGTAGCATCCGATCTTTTCA |  |
| IAA6-R | TGGAGACCAAAACCAGTTGCA |  |
| IAA6-F | GAGACTGGATGCTCGTCGGA |  |
| IBH1-R: | CAAGAGGGCTCTGCTCCATA |  |
| IBH1-F: | GAAGGCTGCGTACGTTTCCA |  |
| PAR2-F | TCTCCTCCGTCTCCATCCTCCG |  |
| PAR2-R | ATCTTTTTTCTCACAACGCTC |  |
| PRE1-R' | CATGAGTAGGCTTCTAATAACGG |  |
| PRE1-F | GTTCTGATAAGGCATCAGCCTCG |  |
| PRE5-R | CATGAGTAAGCTTCTAATCACGG |  |
| PRE5-F | AACGGCGTCGTTCTGATAAG |  |
| PRE6-R | CGGTCACTGAGGTCATCAACCTCTC |  |
| PRE6-F | TCCAACACCTCATCCCTGAACTTCG |  |
| *SAUR15-F* | GTATTGTTAAGCCGCCCATTGG |  |
| SAUR15-R | AAGAGGATTCATGGCGGTCTATG |  |
| SAUR19-F | CTTCAAGAGCTTCATAATAATTCAAACTT |  |
| SAUR19-R | GAAGGAAAAAATGTTGGATCATCTT |  |
| SAUR23-R | ACAAGGAAACAACTCTATCTCTAACT |  |
| SAUR23-F | ATTCAAACTTTCAGACAAAAGAAATGG |  |
| SAUR24-F | GAGATATTTGGTGCCTGTCTCATATTTAAACC |  |
| SAUR24-R | CAAGAAGAAAGAGGAAAAAGGGCTCATC |  |
| SAUR9-R | TTGATCTTCCGAGTCTGGAGTGT |  |
| SAUR9-F' | TGTGATGAAGTCGTCTTTCGTTC |  |
| SAUR68-F | TTACACTGCGGATCAAGTACGC |  |
| SAUR68-R | TTGGTCCTTCTGTGGGAATACC |  |
